# Draft Genome Sequence of the *Serratia rubidaea* CIP 103234^T^ Reference Strain, a Human-Opportunistic Pathogen

**DOI:** 10.1128/genomeA.01340-15

**Published:** 2015-11-19

**Authors:** Rémy A. Bonnin, Delphine Girlich, Dilek Imanci, Laurent Dortet, Thierry Naas

**Affiliations:** a APHP, Bicêtre Hospital, Bactériologie-Hygiene Unit, Le-Kremlin Bicêtre, France; b EA7361 Structure, dynamic, function and expression of broad spectrum β-lactamases, Paris-Sud University, LabEx Lermit, Faculty of Medecine, Orsay, France; c French National Reference Center for Antibiotic Resistance, Carbapenemase-Producing *Enterobacteriaceae*, Le Kremlin-Bicêtre, France; d Molecular Genetics and Hormonology Department, Le Kremlin-Bicêtre, France

## Abstract

We provide here the first genome sequence of a *Serratia rubidaea* isolate, a human-opportunistic pathogen. This reference sequence will permit a comparison of this species with others of the *Serratia* genus.

## GENOME ANNOUNCEMENT

*Serratia* species are Gram-negative rods responsible for human-opportunistic infections. Despite the fact that *Serratia* species are widespread in the environment, they are also encountered in human fecal flora. *Serratia marcescens*, the main representative of *Serratia*, was discovered in the early 19th century by the Italian microbiologist Bizio (1).

Now, the genus comprises 18 species recovered from environment and clinical specimens (http://www.bacterio.net/). Among pathogenic species, *S. marcescens* is the most frequently identified, along with *Serratia liquefaciens*. Infections caused by these organisms are varied, including urinary tract infections (UTIs), endocarditis, and wound and pulmonary infections (1). *Serratia rubidaea*, although rarely recovered from human specimens, is recognized as the fourth common cause of *Serratia*-related infections (1). Infections caused by *S. rubidaea* are mainly reported in patients with severe trauma or with underlying diseases, including sepsis, bacteremia, and UTIs (2–5). *Serratia* spp. may be a source of difficult-to-treat infections, since many of these strains are resistant to β-lactams mediated by the production of chromosomally encoded β-lactamases of either Ambler class C (AmpC-type of *S. marcescens*), Ambler class A (FonA and SFC-1 of *Serratia fonticola*), or Ambler class B (Sfh-I of an environmental *S. fonticola*) (6, 7).

Genomic DNA was extracted using the UltraClean microbial DNA isolation kit (Mo Bio Laboratories) from overnight cultures in LB agar (Bio-Rad, Marnes-la-Coquette, France). Genomic DNA quantification was performed using a Qubit fluorometer (Life Technologies, Carlsbad, CA) and adjusted to 0.2 ng/µl. Library preparation was performed using the Nextera XT DNA sample preparation kit (Illumina, San Diego, CA). Sequencing was performed on an Illumina MiSeq 2000 sequencer with V3 chemistry using 2 × 75-bp paired-end reads.

Illumina sequencing resulted in 4,367,802 reads of an average length of 74.31 nucleotides, giving a total 324,574,495 nucleotides. These generated reads were assembled using Velvet (8) and computed using CLC Workbench version 8.5. Two hundred forty contigs, giving a genome of 4,929,307 bp with a G+C% of 59.3%, were obtained from these raw data and then annotated using the RAST server (http://rast.nmpdr.org/). The RAST system predicted 4,522 coding sequences involved in essential functions, such as cell wall synthesis or RNA/DNA metabolism. One hundred ninety-eight coding sequences (CDSs) were predicted in cell wall and capsule synthesis, with 106 involved in virulence, disease, and defense; 38 involved in cell division and cell cycle; 153 involved in fatty acid and lipid metabolism; 134 involved in the stress response; and 216 and 231 involved in protein and RNA metabolism, respectively.

We hope that this sequence will help for genomic comparisons of the *Serratia* genus.

### Nucleotide sequence accession numbers.

This whole-genome shotgun project has been deposited at DDBJ/EMBL/GenBank under the accession no. LJZP00000000. The version described in this paper is version LJZP01000000.
